# Draft Genome Sequence of the Arsenate-Respiring Bacterium *Chrysiogenes arsenatis* Strain DSM 11915

**DOI:** 10.1128/genomeA.00953-13

**Published:** 2013-11-14

**Authors:** David A. Coil, Jonathon R. Lo, Roger Chen, Naomi Ward, Frank T. Robb, Jonathan A. Eisen

**Affiliations:** University of California Davis Genome Center, Davis, California, USAa; University of Wyoming, Laramie, Wyoming, USAb; University of Maryland School of Medicine, Baltimore, Maryland, USAc; University of California Davis, Department of Evolution and Ecology, Department of Medical Microbiology and Immunology, Davis, California, USAd

## Abstract

Here we present the draft genome sequence of *Chrysiogenes arsenatis* strain DSM 11915, only the second genome sequence from the phylum *Chrysiogenetes*. This strictly anaerobic organism was isolated from arsenic-contaminated gold mine wastewater and respires arsenate or nitrate instead of oxygen. The assembly contains 2,824,977 bp in 22 scaffolds.

## GENOME ANNOUNCEMENT

*Chrysiogenes arsenatis* is a curved, rod-shaped, Gram-negative, strictly anaerobic bacterium that was isolated from arsenic-contaminated mud of a reed bed in the Ballarat Goldfields, Australia (1). Unusually, *C. arsenatis* uses acetate as the electron donor and carbon source, and arsenate as the preferred electron acceptor, producing CO_2_ and arsenite (2). Nitrate or nitrite can also be used as electron acceptors, producing NH_4_. At the time of isolation, this species was the only member of the *Chrysiogenetes* phylum (3) but has since been joined by members of the genera *Desulfurispira* and *Desulfurispirillum* (4, 5). The only other member of this phylum to be sequenced is *Desulfurispirillum indicum* (6). *C. arsenatis* was selected in 2002 as part of a project at the Institute for Genomic Research (TIGR) to sequence the genomes of representatives of the seven phyla of bacteria that at the time had cultured representatives but no available genome sequence. Sanger clone-based sequencing of the genome failed to produce a high-quality assembly, and the project was delayed pending additional sequencing with Illumina technology.

DNA from *C. arsenatis* strain DSM 11915 was extracted at Deutsche Sammlung von Mikroorganismen und Zellkulturen (DSMZ) (German Collection of Microorganisms and Cell Cultures) via lysis with lysozyme and proteinase K, phenol and chloroform extraction, and purification with Prep-A-Gene (7). Sanger libraries (small and medium insert and fosmid) were constructed as previously described (8). Illumina paired-end libraries were made from sonicated DNA using standard Illumina protocols and reagents.

A hybrid genome assembly was generated using MIRA (version 3.9.17) (9), with 33,005 Sanger reads (average length, 1,037 bp) and 4,000,000 Illumina reads (average length, 84 bp) that were randomly subsampled from the 41,433,640 total Illumina reads sequenced. The contigs resulting from this assembly were filtered to remove contigs shorter than 500 bp as well as those with >33% of the average coverage for the assembly. This resulted in 51 contigs which, along with 37,362,734 error-corrected/quality-trimmed Illumina reads generated by the A5 assembly pipeline (10), were used to build 22 scaffolds in SSPACE (11). These Illumina reads were then aligned to the scaffolds using Bowtie (12) to eliminate artifacts in the assembly. One such region (~700 bp) was found, which by BLAST (13) analysis appeared to be a vector sequence and was removed from the assembly. During the creation of GenBank submission files, some contigs were merged based on short overlaps and read pair information, yielding a final collection of 23 contigs. The final assembly contains 2,824,977 bp and has a GC content of 50% and coverage estimates of 12× (Sanger) and 120× (Illumina).

Completeness of the genome was assessed using the Phylosift software (A. Darling, G. Jospin, E. Lowe, E. Matsen, H. Bik, and J. Eisen, submitted for publication), which searches for 40 highly conserved, single-copy marker genes (14). All 40 genes were found in this assembly. Automated annotation was performed using the RAST annotation server (15). *C. arsenatis* DSM 11915 contains 2,592 predicted coding sequences and 43 predicted RNAs.

### Nucleotide sequence accession numbers.

This whole-genome shotgun project has been deposited at DDBJ/EMBL/GenBank under the accession number AWNK00000000. The version described in this paper is version AWNK01000000. Complete Illumina and Sanger reads are available at http://dx.doi.org/10.6084/m9.figshare.799759.

## References

[1] Macy JM Nunan K Hagen KD Dixon DR Harbour PJ Cahill M Sly LI 1996 *Chrysiogenes arsenatis* gen. nov., sp. nov., a new arsenate-respiring bacterium isolated from gold mine wastewater. Int. J. Syst. Bacteriol. 46:1153–1157 8863450 10.1099/00207713-46-4-1153

[2] Krafft T Macy JM 1998 Purification and characterization of the respiratory arsenate reductase of *Chrysiogenes arsenati*s. Eur. J. Biochem. 255:647–653 9738904 10.1046/j.1432-1327.1998.2550647.x

[3] Garrity GM Holt JG 2001 Phylum BV. *Chrysiogenetes* phy. nov., p 421–425 *In* Garrity GM Boone DR Castenholz RW (ed), Bergey's manual of systematic bacteriology, 2nd ed, vol 1 Springer, New York, NY.

[4] Sorokin DY Foti M Tindall BJ Muyzer G 2007 *Desulfurispirillum alkaliphilum* gen. nov. sp. nov., a novel obligately anaerobic sulfur- and dissimilatory nitrate-reducing bacterium from a full-scale sulfide-removing bioreactor. Extremophiles 11:363–370 17242870 10.1007/s00792-006-0048-8

[5] Sorokin DY Muyzer G 2010 *Desulfurispira natronophila* gen. nov. sp. nov.: an obligately anaerobic dissimilatory sulfur-reducing bacterium from soda lakes. Extremophiles 14:349–355 20407798 10.1007/s00792-010-0314-7 PMC2898105

[6] Bini E Rauschenbach I Narasingarao P Starovoytov V Hauser L Jeffries CD Land M Bruce D Detter C Goodwin L Han S Held B Tapia R Copeland A Ivanova N Mikhailova N Nolan M Pati A Pennacchio L Pitluck S Woyke T Haggblom M 2011 Complete genome sequence of *Desulfurispirillum indicum* strain S5(T). Stand. Genomic Sci. 5:371–378 22675586 10.4056/sigs.2425302 PMC3368425

[7] Willis EH Mardis ER Jones WL Little MC 1990 Prep-A-Gene: a superior matrix for the purification of DNA and DNA fragments. BioTechniques 9:92–99 2393578

[8] Wu D Daugherty SC Van Aken SE Pai GH Watkins KL Khouri H Tallon LJ Zaborsky JM Dunbar HE Tran PL Moran NA Eisen JA 2006 Metabolic complementarity and genomics of the dual bacterial symbiosis of sharpshooters. PLoS Biol. 4:e188. 10.1371/journal.pbio.0040188 16729848 PMC1472245

[9] Chevreux B Wetter T Suhai S 1999 Genome sequence assembly using trace signals and additional sequence information, p 45–56 *In* Computer science and biology: proceedings of the German Conference on Bioinformatics. Research Centre for Biotechnology (GBF), Braunschweig, Germany

[10] Tritt A Eisen JA Facciotti MT Darling AE 2012 An integrated pipeline for de novo assembly of microbial genomes. PLoS One 7:e42304 23028432 10.1371/journal.pone.0042304 PMC3441570

[11] Boetzer M Henkel CV Jansen HJ Butler D Pirovano W 2011 Scaffolding pre-assembled contigs using SSPACE. Bioinformatics 27:578–579 21149342 10.1093/bioinformatics/btq683

[12] Langmead B Trapnell C Pop M Salzberg SL 2009 Ultrafast and memory-efficient alignment of short DNA sequences to the human genome. Genome Biol. 10:R25 19261174 10.1186/gb-2009-10-3-r25 PMC2690996

[13] Altschul SF Gish W Miller W Myers EW Lipman DJ 1990 Basic local alignment search tool. J. Mol. Biol. 215:403–410 2231712 10.1016/S0022-2836(05)80360-2

[14] Wu D Jospin G Eisen J Systematic identification of gene families for use as markers for phylogenetic and phylogeny-driven ecological studies of bacteria and archaea and their major subgroups. PLoS One, in press 10.1371/journal.pone.0077033 PMC3798382 24146954

[15] Aziz RK Bartels D Best AA DeJongh M Disz T Edwards RA Formsma K Gerdes S Glass EM Kubal M Meyer F Olsen GJ Olson R Osterman AL Overbeek RA McNeil LK Paarmann D Paczian T Parrello B Pusch GD Reich C Stevens R Vassieva O Vonstein V Wilke A Zagnitko O 2008 The RAST server: rapid annotations using subsystems technology. BMC Genomics 9:75 18261238 10.1186/1471-2164-9-75 PMC2265698

